# Allometric scaling of intraspecific space use

**DOI:** 10.1098/rsbl.2015.0673

**Published:** 2016-03

**Authors:** Carolyn M. Rosten, Rodolphe E. Gozlan, Martyn C. Lucas

**Affiliations:** 1 Norwegian Institute for Nature Research (NINA), Høgskoleringen 9, 7034 Trondheim, Norway; 2 School of Biological and Biomedical Sciences, Durham University, Science Laboratories, South Road, Durham DH1 3LE, UK; 3 Institut de Recherche pour le Développement, Unité Mixte de Recherche Biologie des Organismes et Ecosystèmes Aquatiques (IRD 207, CNRS 7208, MNHN, UPMC) Muséum National d'Histoire Naturelle, Paris Cedex 75231, France

**Keywords:** allometry, home range, behaviour, metabolic ecology

## Abstract

Allometric scaling relationships enable exploration of animal space-use patterns, yet interspecific studies cannot address many of the underlying mechanisms. We present the first intraspecific study of home range (HR) allometry relative to energetic requirements over several orders of magnitude of body mass, using as a model the predatory fish, pike *Esox lucius*. Analogous with interspecific studies, we show that space use increases more rapidly with mass (exponent = 1.08) than metabolic scaling theories predict. Our results support a theory that suggests increasing HR overlap with body mass explains many of these differences in allometric scaling of HR size. We conclude that, on a population scale, HR size and energetic requirement scale allometrically, but with different exponents.

## 1. Introduction

Space-use patterns are a fundamental aspect of animal ecology, with implications including resource acquisition, behavioural interactions (e.g. mate searching, competition) and human–wildlife interactions [1–3]. Home range (HR), or the area used by an animal for daily activities [4], is an empirical measure of space use, known to increase allometrically, since larger bodied individuals require more space to meet their energetic requirements [5]. Substantial research has been conducted into the scaling relationships between body mass, metabolic rate and space use at an interspecific level (e.g. [6]). However, the relationships are not always straightforward, so the underpinning ecological mechanisms remain poorly understood [6] and the direct metabolic interaction with space-use strategy remains elusive.

Early work proposed a directly proportional relationship between HR size and metabolic rate, suggesting that both scaled with body mass (*M*) at a rate close to *M*^0.75^ [7]. This led to the conclusion that HR size was a direct reflection of energetic requirement, though recent studies demonstrate the ¾ power law of metabolic rate scaling to be far from universal [8]. Empirical studies found a significantly steeper increase in HR size relative to energetic requirements than the theoretical *M*^0.75^. One leading explanation for the discrepancy is the ‘gas model’ of Jetz *et al.* [9], which predicts the frequency of interaction, spatial overlap and loss of resources using an equation taken from physics for collisions among gas particles to predict the frequency of interactions of neighbours. With this model, they predicted that while HR size increases at a rate of *M*^0.75^, daily travel distance for foraging within the HR increases only at a rate of *M*^0.25^ [8], supported by empirical studies [10]. Consequently, larger individuals cover the full extent of their HR less often, leading to lesser expulsion of competitors and thus greater overlap of HRs. Increased resource sharing ensues, with a related requirement for greater relative HR size at larger body size. Further detail is given in [9].

Interspecific animal space-use studies are complicated since factors other than metabolic requirements may be drivers of variability in space-use traits, e.g. latitude or carnivory/herbivory. Marked variation in space use may also occur within species [11], for example, covarying with habitat quality. High-quality habitats are expected to result in small HRs, whereas low productivity habitats are associated with larger HRs [12]. Though within intraspecific studies there will still be factors that could modify space use, investigating intraspecific scaling of HR size within a single, highly size-variable species enables additional consideration of individual behaviour and physiology influences not otherwise possible.

Fishes are the only vertebrate group in which an individual's life history can span eight orders of magnitude in body size [13]. Pike (*Esox lucius*), a freshwater predatory fish, is an ideally suited species for examining scaling of individual space use since, within a single species where juvenile and adult body form and habitat use are similar, pike body size spans several orders of magnitude.

In this study, we address two key questions on space use, employing a detailed dataset of pike space use. First, based on allometric scaling relationships of key space-use attributes, we test whether these variables follow predictions made by theory. Second, we explore some underpinning potential drivers. Specifically, we predict that HR size will scale at a rate greater than required solely by energetic requirements and that daily travel distance will scale at a substantially lower rate.

## 2. Material and methods

The study was conducted on the River Frome, England (50°419 N; 2°119 W). We measured individual summer HR and mean daily travel distance using radio telemetry of 43 pike ranging in body mass (*M*) from 7 to 12 060 g between June and September 2001–2005. Fish were located at dawn, midday and dusk every day over a 13 day period, resulting in standard summer HR datasets of 39 locations per fish. Armstrong *et al.* [14] published a scaling relationship of metabolic rate of pike with body mass and we used the log-transformed data from all individuals in that study to generate confidence intervals around the scaling exponent and test for a significant difference between the metabolism and HR scaling exponents. Metabolic data were collected at 15°C, while average summer water temperatures of the River Frome varied between 15 and 17.5°C [15]. Linear regression applied to log–log transformed data (*M* versus *K*_99_, *M* versus mean daily travel distance and *M* versus metabolic rate) gave coefficients of the slopes around which confidence limits were generated. This enabled significance testing of the slopes of the different relationships. The back-transformed equation was plotted onto the arithmetic data for assessment of the fit of the power law model on the arithmetic scale. For more information, see the electronic supplementary material, materials and methods. Statistical analysis was conducted in R and Minitab.

## 3. Results

**Figure 1.**
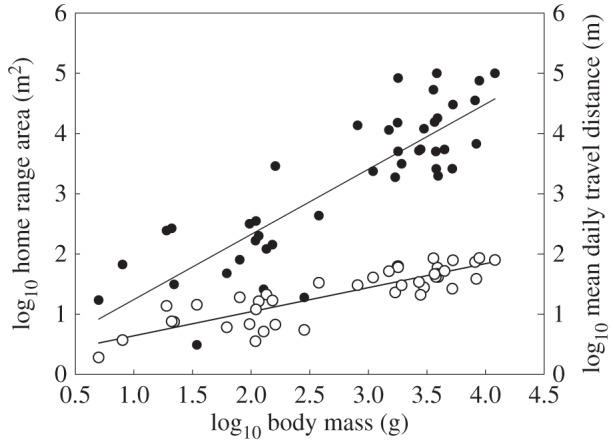
Allometric scaling on the geometric scale between body mass (*M*, g) and both home range area (m^2^) (HR, filled circles), log_10_ HR = 0.16 + 1.08 log_10_ *M* and mean daily travel distance (DTD, open circles) log_10_ DTD = 0.24 + 0.40 log_10_ *M* in pike, *Esox lucius*.

**Figure 2.**
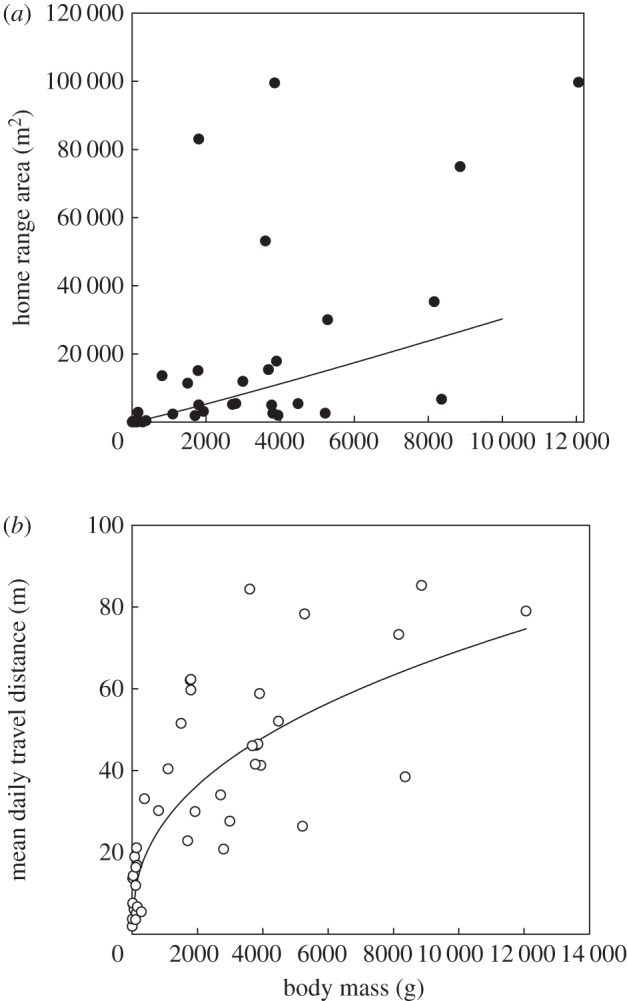
Allometric scaling on the arithmetic scale between body mass (*M*, g) and (*a*) home range area (HR, m^2^) HR = 1.45 *M*^1.08^ and (*b*) mean daily travel distance (DTD, m) DTD = 1,74 *M*^0.40^ in pike, *Esox lucius*.

Both individual HR and mean daily travel distance showed strong allometric scaling (figures 1 and 2). Individual HR size scaled with an exponent of *M*^1.08^ (linear regression of log-transformed data, *p* < 0.001), significantly higher (*p* < 0.05) than *M*^0.75^ predicted by McNab [7] and *M*^0.80^ previously measured for pike standard metabolic rate [14]. Thus, the trend of HR increasing with body mass more rapidly than predicted by metabolic needs alone, observed in interspecific studies, is demonstrated here for a single species. Mean daily distance travelled scaled as *M*^0.40^ (figures 1 and 2; linear regression of log-transformed data, *p* < 0.001), increasing at a much lower relative rate than HR size, indicating a reduced HR traversing frequency for larger individuals. This follows the prediction of the Jetz *et al.* [9] model that a lower allometric increase in daily travel distance leads to a lower extent of traversing the full HR. Presentation of the power functions plotted on the arithmetic scale is provided in figure 2, while figure 1 demonstrates linearity of the relationship in the log domain.

## 4. Discussion

Our results suggest that increased HR size leads to greater HR overlap owing to lesser patrolling of the full range area. HR scaling patterns observed in interspecific studies were present within a single species. The two scaling studies of standard metabolic rate in pike found exponents that fell within the higher scaling range typical for teleost fishes [13] (0.80 and 0.82 in [14] and [16], respectively). HR in pike scales with body size at a significantly greater rate than these two higher species-specific exponents, as well as the 0.75 exponent commonly referred to in many interspecific metabolism scaling studies [8].

HR establishment is the result of resource availability, individual behaviour and physiology, and interactions both within and between species (e.g. [3]). We have shown that HR increase with larger body mass is greater than basic energetic requirements might suggest. Jetz *et al.* [9] proposed that spatial overlap and loss of resources to neighbours were driving the steep allometric increase of HR. Our results support their model, since daily distance travelled increased at a rate of approximately 0.4 compared to a HR scaling exponent of over 1 (figure 1). Thus, larger individuals covered 60% less ground relative to their body size than did smaller individuals. While pike are not territorial and do inhabit overlapping HRs [17], they are known to adapt their behaviour and reduce attack frequencies and prey consumption rates in the presence of conspecifics [18]. Thus, it seems likely that with increasing spatial overlap between conspecifics and a lack of territorial behaviour, there is a need for larger HRs than would be predicted based on metabolic needs alone.

Scaling down to intraspecific studies introduces some challenges from population and individual scale traits such as behavioural syndromes [19]. However, despite these potentially masking factors, the patterns demonstrated interspecifically were also clearly represented within a species, thus opening the opportunity for exploring the mechanisms behind the patterns. Further work with model species exhibiting prolonged growth over several orders of magnitude of body mass while maintaining relatively stable body morphology, as occurs in many post-hatchling reptiles and post-larval fishes, will enable deeper exploration of the mechanisms behind allometric scaling of space use.

We conclude that, on a population scale, an allometric relationship does exist between HR size and energetic requirement, despite individual variation in factors such as resource distribution, behaviour, physiology and interaction.

## Supplementary Material

Rosten et al Supporting Material.docx

## Data accessibility

Data are available from the Dryad Digital Repository http://dx.doi.org/10.5061/dryad.n7b7d.
