# Supplementary material for: Allometric scaling of intraspecific space use
Source: Biol Lett. 2016 Mar;12(3):20150673. doi: 10.1098/rsbl.2015.0673 (PMC4843214; doi:10.1098/rsbl.2015.0673)
Supplement: Rosten et al Supporting Material.docx [file rsbl20150673supp1.docx]

**Allometric Scaling of Intraspecific Space Use**

**Supplementary Material**

Carolyn M. Rosten,^1,2,*^ Rodolphe E. Gozlan,^3^ Martyn C. Lucas,^2^

^1^ Norwegian Institute for Nature Research (NINA), Høgskoleringen 9, 7034 Trondheim, Norway

^2^ School of Biological and Biomedical Sciences, Durham University, Science Laboratories, South Road, Durham DH1 3LE, UK.

^3^ Institut de Recherche pour le Développement, Unité Mixte de Recherche Biologie des Organismes et Ecosystèmes Aquatiques (IRD 207, CNRS 7208, MNHN, UPMC) Muséum National d’Histoire Naturelle, 75231 Paris Cedex, France

^*^Author for correspondence: [Carolyn.Rosten@nina.no](mailto:Carolyn.Rosten@nina.no)

Material and methods

(a) Study site

The study was conducted on the River Frome, England during summer when fish are feeding and growing rapidly. Most fish locations were collected within a 2 km stretch of river (50°41’ 52” N; 2°11’39” W), although fish were free to move throughout the river and were also tracked outside this stretch.

(b) Tagging

Forty three fish were collected by electric fishing or angling according to their size and location. Pike ranging in body mass from 7 to 12 060 g were tagged. Three radio tag types were used for different size classes of fish (table S1). Tag mass as a proportion of fish body mass was 1.82% ± 1.79 (*mean* ± *SD*) for fish with PIP-3 tags, 0.32% ± 0.21 for fish with TW-4 tags and 0.70% ± 0.33 for fish with TW5 tags. These tag mass to fish mass ratios are considered to be appropriate for telemetry studies without excessive tag burden effect [[1](#_ENREF_1)]. Fish were anaesthetized in 2-phenoxyethanol (1:1000) then length and mass (*M*) were taken prior to tag attachment. Details for attachment of TW-5 and TW-4 tags are given in [[2](#_ENREF_2), [3](#_ENREF_3)]. Due to the short battery life and attachment period and also the sensitivity of very small pike to tight external attachment, PIP-3 tags were attached using a single suture through the musculature just posterior to the dorsal fin [[4](#_ENREF_4)]. Ten days recovery was allowed prior to the commencement of data collection [2].

(c) Telemetry and Home Range Estimation

Telemetry was carried out between June and September 2001 to 2005. This period was selected to avoid spawning activity, which occurs in the spring. Fish were located at dawn, midday and dusk every day over a consecutive 13 day period, resulting in standard summer home range data sets of 39 locations per fish. Thus home ranges reflected the full area used by the individual in a given season (39 locations were chosen since home range size stabilised after inclusion of this number of locations) [[4](#_ENREF_4), [5](#_ENREF_5)]. Fish location data were analysed to generate 99% kernel probability distributions (*K*_99_) to produce home ranges in m^2^ and clipped to within the river outline using RANGES 8 (Anatrack Ltd, Wareham, UK) following procedures developed during previous studies of the pike population [[6](#_ENREF_6)]. Daily travel distance was calculated from the minimum linear distance (following the river’s midline) between two pike locations [[5](#_ENREF_5)]. The mean of all daily travel distances over the 13 day tracking period was calculated.

(d) Data Analysis

Armstrong et al [7] published the scaling relationship of metabolic rate of pike with body mass from log-transformed values and we used the metabolic rate and mass data for all individual pike used to derive that scaling coefficient. Linear regression was performed on log-transformed *M* vs *K*_99_, *M* vs mean daily travel distance and *M* vs metabolic rate (the latter using original data from Armstrong et al [7] since confidence limits were not provided in the published paper). 95% confidence limits were calculated about the slopes and these were assessed for significant differences between slopes (i.e. where confidence limits did not cross, e.g. [[7](#_ENREF_7)]). The allometric relationship of body size with both home range area and mean daily travel distance was plotted on the arithmetic scale to enable inspection of the fit of the back transformed power functions (figure 2) [[8](#_ENREF_8)].

**Table S1**. Details of the three radio tag types and tagging procedure used for telemetry of pike of different size classes.

| Tag | Dimensions | | | Tag lifetime | Fish size range |  | Tag attachment | Ref. |
| --- | --- | --- | --- | --- | --- | --- | --- | --- |
|  | Length (mm) | Diameter (mm) | Weight in water (g) |  |  | No. fish |  |  |
| PIP 3 (Biotrack Ltd, Wareham, UK) | 7 | 4 | 0.4 | 3 - 4 weeks | 10-25 cm  (7-115 g) | 10 | External | - |
| TW-4 (Biotrack) | 10 | 6 | 0.7 | 5 months | 26 -55 cm  (116-1500 g) | 10 | External | [[2](#_ENREF_2)] |
| TW-5 (Biotrack) | 80 | 16 | 22 | 3 years | ≥ 56 cm  (≥ 1500 g) | 22 | Internal | [[3](#_ENREF_3)] |

References

[1] Cooke, J.J., Hinch, S., Lucas, M.C. & Lutcavage, M. 2012 Biotelemetry and biologging. In *Fisheries Techniques 3rd ed* (eds. A. Zale, D. Parrish & T. Sutton). Bethesda, Maryland, American Fisheries Society.

[2] Beaumont, W.R.C., Clough, S., Ladle, M. & Welton, J.S. 1996 A method for the attachment of miniature radio tags to small fish. *Fish. Manage. Ecol.* **3**, 201-207.

[3] Beaumont, W.R.C., Cresswell, B., Hodder, K.H., Masters, J.E.G. & Welton, J.S. 2002 A simple activity monitoring radio tag for fish. *Hydrobiologia* **483**, 219-224.

[4] Knight, C.M. 2006 Utilisation of off-river habitats by lowland river fishes. PhD Thesis, Durham University.

[5] Hodder, K.H., Masters, J.E.G., Beaumont, W.R.C., Gozlan, R.E., Pinder, A.C., Knight, C.M. & Kenward, R.E. 2007 Techniques for evaluating the spatial behaviour of river fish. *Hydrobiologia* **582**, 257-269.

[6] Knight, C.M., Kenward, R.E., Gozlan, R.E., Hodder, K.H., Walls, S.S. & Lucas, M.C. 2009 Home-range estimation within complex restricted environments: importance of method selection in detecting seasonal change. *Wildl. Res.*

[7] Glazier, D.S. 2008 Effects of metabolic level on the body size scaling of metabolic rate in birds and mammals. *Proceedings of the Royal Society: B* **275**, 1405-1410. (doi:10.1098/rspb.2008.0118).

[8] Anscombe, F.J. 1973 Graphs in statistical analysis. *American Statistician* **27**, 17-21.
